# Concise Review: Criteria for Chamber‐Specific Categorization of Human Cardiac Myocytes Derived from Pluripotent Stem Cells

**DOI:** 10.1002/stem.2649

**Published:** 2017-06-27

**Authors:** Christopher Kane, Cesare M. N. Terracciano

**Affiliations:** ^1^ Imperial College London, National Heart and Lung Institute, Hammersmith Campus, BHF Centre for Regenerative Medicine London United Kingdom

**Keywords:** Induced pluripotent stem cell‐derived cardiac myocytes, Induced pluripotent stem cells, Heart, Chamber specificity, Maturation, Heart disease models, Cardiac toxicology models

## Abstract

Human pluripotent stem cell‐derived cardiomyocytes (PSC‐CMs) have great potential application in almost all areas of cardiovascular research. A current major goal of the field is to build on the past success of differentiation strategies to produce CMs with the properties of those originating from the different chambers of the adult human heart. With no anatomical origin or developmental pathway to draw on, the question of how to judge the success of such approaches and assess the chamber specificity of PSC‐CMs has become increasingly important; commonly used methods have substantial limitations and are based on limited evidence to form such an assessment. In this article, we discuss the need for chamber‐specific PSC‐CMs in a number of areas as well as current approaches used to assess these cells on their likeness to those from different chambers of the heart. Furthermore, describing in detail the structural and functional features that distinguish the different chamber‐specific human adult cardiac myocytes, we propose an evidence‐based tool to aid investigators in the phenotypic characterization of differentiated PSC‐CMs. Stem Cells *2017;35:1881–1897*

**Significance Statement**

The concepts of development and maturation of in vitro generated heart cells are often confused and misinterpreted, particularly when a cell group is thought to resemble a specific heart chamber subpopulation. With an enormous increase in the use of PSC‐cardiac myocytes for regeneration strategies, disease modeling, and drug testing, this field is both very topical and ever expanding. In this article, not only we describe all the current knowledge on the criteria that distinguish cardiac myocytes from different heart chambers but we also provide a phenotypic tool that can aid in the categorization of cardiac myocytes derived from PSCs.

## Introduction

The sophisticated and highly specialized anatomy of the heart is designed as such to produce co‐ordinated and continuous flow of blood through the pulmonary and systemic circulations, tirelessly contracting under rigorous mechanical demands for the entirety of the human lifespan. The complex mechanical and electrical functions of the heart originate predominantly from a single cell type—the cardiomyocyte (CM)—whose properties become specialized to particular anatomical regions to meet their unique functional requirements.

**Figure 1:**
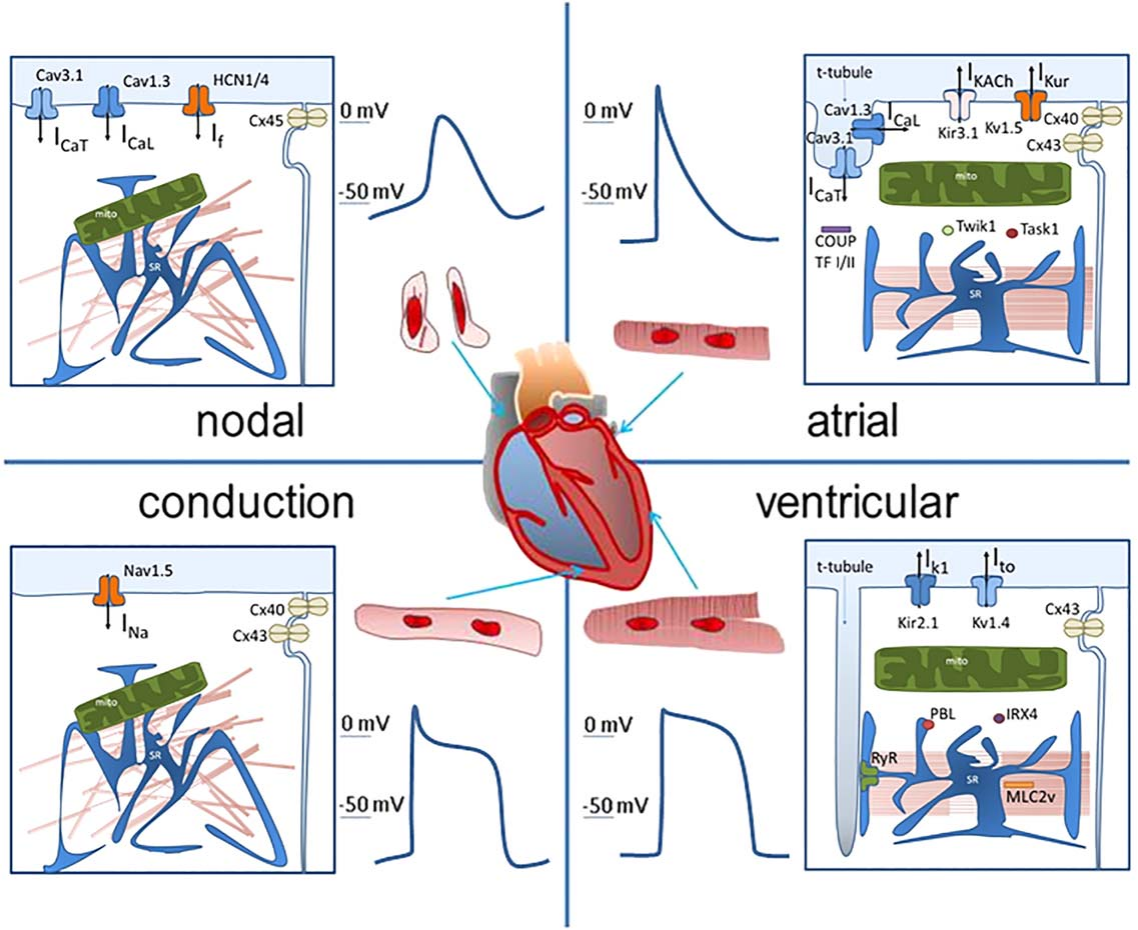
This figure shows the most commonly accepted features that identify myocytes from nodal tissue, the conduction His‐Purkinje system, working atrial, and ventricular myocardium. Each panel shows the gross morphological structure of the cells, the action potential profile, and the subcellular structure with the most common molecular markers associated with the specific function of the region. The clear distinction between regions of the heart is for classification purposes only, as cardiac myocytes from different regions show a vast spectrum of functional and structural properties, and there is significant overlap in these properties between different regions. Abbreviations: COUP, chicken ovalbumin upstream promoter; HCN, Hyperpolarization‐activated cyclic nucleotide–gated; MLC, myosin light chain; TF, transcription factor.

The CMs of the ventricles are the workhorses of the heart, highly developed muscle cells dense with contractile elements, mitochondria, and efficient calcium handling machinery that allow them to produce the high levels of force and pressures required to perfuse the systemic circulation. Atrial CMs, while also primarily dedicated to contraction, are smaller and less well organized compared with their ventricular counterparts, as lower atrial pressures and the low resistance of ventricular filling mean they do not need to produce the same levels of force. The cells of the His‐Purkinje system lack much of the contractile apparatus and are instead dedicated to the transmission of electrical impulses throughout the ventricles. While all cardiac tissue is conductive, the ability of Purkinje fibers to rapidly convey electrical signals from the atria to the apex of the ventricles is crucial for the correct, sequential, and co‐ordinated contraction of the heart necessary for efficient ventricular filling and emptying. Finally, and perhaps most distinctive are the cells of the sino‐atrial (SA) and atrio‐ventricular (AV) nodes. The unique cyclical nature of their electrical activity means that these cells are the origin of the heartbeat itself, rhythmically generating depolarizations that ultimately spread through the conduction system and ultrastructure of the heart to trigger the cardiac cycle (Fig. 1).

While decades of experimentation with cells enzymatically isolated from adult hearts has provided a detailed description of the underlying electrical, mechanical, and genetic factors that produce these different CM phenotypes, ultimately the chamber‐specific features of these cells are categorized and referred to on the basis of their anatomical origin. As such, the exercise of trying to categorize CMs on the basis of nonanatomical parameters may seem futile. However, the advent of cardiac myocytes differentiated from pluripotent stem cells [(PSC), embryonic stem cells (ESC) or induced PSCs (iPSCs)] has brought this issue back into the limelight as PSC‐derived CMs have never been in a beating heart and therefore have no anatomical origin to reference. In addition, their development is entirely carried out in a reductionist environment in vitro and therefore even their specific developmental history is artificial and cannot supply information regarding their fate.

In this article, we aim to explain why the issue of chamber‐specific categorization of cardiac myocytes is important in the new and exciting field of PSC‐CMs for physiology, pharmacology, and regenerative studies as well as discuss previous strategies used to categorize cardiac myocytes into atrial, ventricular, and of the conduction system in the absence of a known location in the heart. We describe in detail the structural and functional features that distinguish the different chamber‐specific human cardiac myocytes and from this propose a standard set of criteria that may be used for such categorization in the future.

## Chamber‐Specific Categorization of PSC‐Cardiac Myocytes

PSC‐cardiac myocytes are one of the most interesting cardiovascular research platforms of last decade. Derived from two sources, ESCs and iPSCs, these cells represent a limitless supply of human CMs with the potential to address needs unmet by current cell and animal models, eliminating the pitfalls of differential interspecies physiology and improving the translation of experimental findings into clinical practice. iPSC‐derived CMs are particularly interesting as they are derived from adult somatic cells and retain the genetic characteristics of their donor, opening up potential applications in modeling familial cardiac disease, personalized drug screening, and autologous cell therapies.

Several protocols for differentiation of both ESCs and iPSCs into CMs have been described, with strides made in achieving both the highest purity and quality cells 1, 2, 3. While discussion of these protocols is beyond the scope of this article, there is strong evidence that they are able to produce robust cardiac differentiation of PSCs. Broadly, these differentiations are unguided; that is to say they do not aim to produce CMs of a particular chamber type and are commonly reported to contain various proportions of “nodal‐like,” “atrial‐like,” and “ventricular‐like” cells 4, 5, 6, 7. The enrichment, therefore, of a particular chamber‐like cell type in these cultures will require additional steps involving cell selection 8, 9 or by providing additional developmental cues 10, 11. More details of these protocols are reported later. What is clear is that in many areas there is a strong demand for PSC‐derived CMs with more well defined properties, particularly in terms of specific chamber‐like phenotypes.

A relevant field where selected chamber‐specific PSC‐derived CMs would be desirable is that of disease modeling. The ability to obtain myocytes with a specific phenotype may help to detect more specific consequences of the disease, which may not be visible when a mixed population is used. Familial cardiomyopathies may affect myocyte subtypes differently, as a consequence of differential protein expression in different chambers (see details below) and future studies should consider this aspect.

Two other fields of research are particularly important: the use of chamber‐specific PSC‐CMs in pharmacotherapy and in cell therapy.

### Chamber‐Specific PSC‐CMs in Pharmacotherapy

The weakness of recombinant cell lines and animal models in the preclinical screening of pharmacological agents has long been appreciated. With the average cost of bringing new agents to market spiraling toward $3 billion 12, while up to 90% of promising compounds fail when tested in human trials 13, the need to develop screening tools that are more relevant to human physiology is great. While the question of how much more representative PSC‐CMs are of human physiology than some animal models is still an open one 14, many feel these cells have the potential to fulfill this unmet need in the development of novel pharmacotherapies 15, 16, 17.

When it comes to the use of chamber‐specific PSC‐CMs, one significant example is in the development of therapies for atrial fibrillation (AF). AF is one of the most common forms of arrhythmia, with a global incidence of over 33 million people 18 as of 2014. In the US alone, the annual cost of AF approaches $26 billion 19, with patient numbers expected to double by 2050 20. Current therapeutic approaches involve a combination of anti‐arrhythmic drugs and rate control through the use of implanted devices. While noninvasive management is preferable from both the point of patient safety and cost, current pharmacotherapies risk substantial side effects such as the induction of ventricular proarrhythmia which may add to the burden of patient management and reduce survival outcomes 21. The answer to this has been a concerted effort in recent years to develop drugs with greater atrial‐selectivity, targeted at ion channels that are uniquely or preferentially expressed in atrial CMs 22.

PSC‐CMs offer a potential platform that would not only express relevant atrial targets for pharmacotherapy, but also an appropriate context in terms of global electrophysiological and functional properties lacking in techniques such as recombinant cell lines that may allow for more faithful assessment of novel compounds. As such, the generation of chamber‐specific PSC‐CMs would be a great advantage in the development of novel targeted therapies.

### Chamber‐Specific PSC‐CMs in Cell Therapy

As the number of people dying annually from myocardial infarction has decreased due to advances in the delivery of interventional therapy, so too has the number of people living with damaged myocardium increased. One potential application of PSC‐CMs is in cell therapy, replacing dead or damaged myocardial tissue, improving cardiac function and preventing decline into heart failure. Numerous clinical trials have been performed injecting stem cell‐derived CMs into damaged hearts and have produced variable results 23, 24. Trials that have reported positive findings such as long term improvements in ejection fraction and reduction in scar size have been unable to demonstrate true engraftment of new CMs, attributing changes to paracrine effects 25, 26. While to date cell therapy has been shown to be both feasible and safe there are concerns that, as techniques improve and functional engraftment of stem cell‐derived CMs is achieved, this may not remain the case.

There are substantial electrophysiological differences between PSC‐CMs and adult CMs, reviewed extensively elsewhere 27, 28, 29. PSC‐CMs have been demonstrated to be able to form gap junctional connections with native CMs, and the electrophysiological mismatch between the two poses a significant risk of arrhythmia 30, 31. While this is partly an issue of the relative immaturity of PSC‐CMs compared with adult myocytes, it is also the case if one considers the chamber‐specificity of the cells being introduced to the myocardium. Engraftment of nodal‐like PSC‐CMs into ventricular tissue risks introducing abnormal automaticity while atrial‐like and other cell types, with substantially different action potential (AP) durations and conduction velocities to the surrounding cells, may produce a heterogeneous arrhythmogenic substrate. As such being able to produce a cell population enriched or exclusively comprised of cells of a particular chamber phenotype would go some way to alleviate the risks of this form of cell therapy.

This ability to establish electronic interaction with native tissue also opens up further applications of these cells. Pacemaker dysfunction represents a substantial burden of cardiovascular disease. The development of sophisticated implantable cardiac devices has delivered ever greater management of these conditions; however, with risks of infection and device failure, the requirement of maintenance as well as a lack of responsiveness to the body's autonomic system makes them a less than perfect solution 32.

Previous attempts to generate a biological pacemaker have included xenografted nodes 33 or nodal cells 34 and the manipulation of native cells to express nodal properties through gene editing 35. A significant advance in this field was reported recently by Protze and coworkers, demonstrating the ability of PSC‐derived sinus atrial node‐like cells to successfully pace host tissue when engrafted into rat hearts 36. Given that the potential output of such an approach may be an adaptable, responsive, functioning, and autologous node, this represents an exciting application of chamber‐specific PSC‐derived CMs.

## Approaches to Assigning Chamber Type

### AP Morphology

One of the most widely recognized features of CMs from different chambers of the heart is the morphology of their AP. Differential expression of several ion channels across the chambers of the heart results in distinctive AP morphologies in different heart chambers 37. As such, AP morphology is commonly used to identify whether a cell has a particular chamber bias.

**Figure 2:**
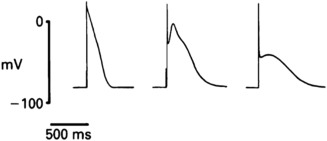
Human atrial action potentials (APs) have a spectrum of different morphologies. These APs were all recorded from human atria (adapted from 85).

**Figure 3:**
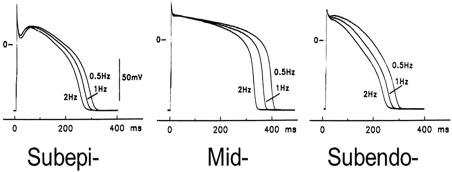
Variability of morphology of human action potentials from different regions of the ventricular wall (adapted from 105).

There are several limitations to this approach. First, while the textbook definitions of chamber‐specific AP waveforms are fairly distinct, these are in many ways caricatures of a much broader spectrum of morphologies; this masks the variety of AP waveforms that can be found throughout the atria and across the ventricular wall (Figs. 2, 3) 40, 41, 42. These ranges make it difficult to develop robust quantitative criteria that can delineate between cell types; how does one distinguish between an atrial CM with a long AP and a ventricular CM with a short AP?

PSC‐CMs themselves also pose a challenge to classification on this basis. While numerous reports have detailed the presence of nodal‐like, atrial‐like, and ventricular‐like APs in unguided differentiations of iPSC‐CMs 4, 6, 7, 43, recent work by our group and others has shown that the AP morphologies of iPSC‐CMs do not robustly discriminate between multiple subpopulations of cells 44, 45, 46, 47, 48. While the question of whether different cell populations are present remains to be answered, if it is the case then they are not sufficiently distinct to be classified on the basis of their AP properties. A recent study using a genetically encoded membrane voltage indicator targeted to chamber‐specific molecules has been able to discriminate between subgroups in a mixed population of myocytes 8. Unfortunately, the specificity of these molecules for a cardiac region is often questionable (e.g., the case of HCN for nodal myocytes 49).

Furthermore, we and others have shown that the AP of iPSC‐CMs, rather than fixed, is highly plastic and is heavily dependent on culture environment. Compared with confluent culture, iPSC‐CMs cultured at low density, as is necessitated by microelectrode techniques commonly used to assess AP properties, have longer and more heterogeneous APs with decreased expression of ion channels that give rise to *I*_Ks_ and *I*_K1_, critical repolarizing currents that are already severely underrepresented in these cells compared with adult 44, 50. This confounding factor, together with differences in AP morphology existing between isolated cells and multicellular preparations 51, suggests that AP morphology is not a suitable indicator of overall cell properties. The observation that AP morphology in PSC‐CMs is strongly dependent on the duration of culture 43 further supports this concept.

### Gene and Protein Marker Expression

In addition to electrophysiological properties, there are a wide range of genes and proteins identified as uniquely or preferentially expressed in different anatomical regions of the heart and are therefore used as markers to identify the origin or chamber type of cells. As an example, the channel subunit contributing to the ultra‐rapid delayed rectifier potassium current (*I*_Kur_) Kv1.5, is uniquely expressed in CMs of the atria and contributes to their classic notched AP morphology, and as such is a commonly used marker of atrial CMs 52, 53.

The use of identifying markers of this nature suffers from an important limitation. Often markers of this nature display both anatomical and temporal variability. The myosin light chain (MLC) isoform MLC2V is often ascribed as a general ventricular marker, but is expressed differentially between the right and left ventricles and substantially reduced in areas other than the ventricular free walls in mice 54. In humans, MLC2V may be more homogeneously distributed in both ventricles with low expression in atria, but its expression in nodal tissue cannot be excluded 55. The expression of MLC2A and the Ca^2+^ binding protein S100A1 is fairly well restricted to the atria and ventricles, respectively, in adulthood, but are coexpressed in both chambers during development 54, 55, 56. This temporal dependence of expression represents a particular difficulty for PSC‐CM identification as these cells are often described as being at an embryonic‐fetal stage of development, and as such predicting expression of developmentally dependent markers in these cells may be challenging. A recent example where, using single cell analysis, the nodal marker HCN was found in all the cells of a mixed population of PSC‐CMs 49 confirms that these markers alone are insufficient to determine chamber specificity of CMs.

## Chamber‐Specific Features of Human CMs

To develop a template that can be used to assess the chamber‐specific nature of PSC‐derived CMs, we have below compiled a description of the chamber‐specific structural, electrophysiological, and genetic features of adult human CMs as they are currently understood. While the various subpopulations of CMs can be drawn into numerous categories, we have limited our consideration for the purpose of this exercise to four broad anatomical groups: nodal, conduction, atrial, and ventricular CMs. Where data obtained from human CMs are scarce or unavailable, we have included that observed in animal species whose physiological properties more closely resemble that of human. On that basis, rat and mouse data have been excluded. All relevant electrophysiological parameters reported were obtained with 1–2 Hz stimulation at 37°C.

Table 1 gives a summary of the general distinctive features observed in the four main populations of cardiac myocytes.

**Table 1:** Summary of specific features of myocytes from different regions

Nodal	Small and irregularly shaped myocytes with no t‐tubular network and limited sarcomeric organization. Their intrinsic activity is a product of both the presence of *I*_f_ and SR Ca^2+^ leak. They show action potentials (APs) with a MDP of around −65 mV and a slow *V*_max_ of about 4–5 V/s. APA is ∼80 mV and APD90 ∼150 ms. They express high levels of HCN1, HCN4, CaV3.1, CaV1.3, and Cx45 and low or absent levels of Cx40, Cx43, NaV1.5, Kir2.1, HERG, Kv4.3, and Kv1.5.
Conduction	Purkinje cells are large cylindrical myocytes with poor subcellular organization and spontaneous beating activity. They have a MDP of ∼–85/–90 mV, APD50 of ∼220 ms and APD90 of ∼300 ms. They express very similar arrays of genes compared with the atria and ventricles but are characterized by low expression of KChip2, SERCA2a, NCX1, and RYR2.
Atrial	These striated, rod shaped muscle cells display a highly organized sarcomere structure and limited t‐tubular network. They show a broad range in AP morphologies with MDP between −65 mV and −80 mV, a *V*_max_ of approximately 200 V/s, APA of approximately 100 mV and a variable duration, with APD50 between 25 and 200 ms and APD90 between 200 and 400 ms. AP duration is sensitive to *I*_K,Ach_ and *I*_Kur_. Spontaneous activity is extremely low. Atrial cells show abundant expression of Cx40 and 43, Kv1.5 and Kir3.1, Cav1.3, Cav3.1, CaVα2δ2, NaVβ1, TWIK1, TASK1, NaV1.5, KV4.3, sarcolipin, COUP‐TFI, and II.
Ventricular	Ventricular cardiomyocytes are large, rod‐shaped striated muscle cells with clear sarcomere organization and well‐developed t‐tubular structure. There are several possible AP morphologies with a MDP of∼–75 to −90 mV, APA of ∼100 mV, AP50 of ∼200–300 ms and APD90 of ∼250–450 ms. In the absence of stimulation ventricular cells are quiescent. Ventricular markers are Kir2.1, RYR2, phospholamban, Kv1.4, MLC2V, IRX4 with low Cx40, Kv1.5 and Kir3.1, Cav1.3, Cav3.1, S100A1.

Abbreviations: SAP, action potential, APA, action potential amplitude; MDP, maximum diastolic value, SR, sarcoplasmic reticulum.

## Nodal Myocytes

These are the myocytes found in the sinoatrial node (SAN) and the atrioventricular node (AVN).

### Cell Morphology

The cellular component of the SAN is highly heterogeneous in structure. Three distinct populations of myocytes have been reported: the pacemaker (P) cells, working myocardial cells, and transition cells with intermediate features 57. Human P cells are polyhedral, though may appear elongated in isolation, with a maximum diameter of 5–10 μm. They present a pale aspect due to an “empty” cytoplasm compared with cells of the working myocardium, owing to a limited number of sarcomeres, which are variable in length and orientation 58. P cells do not display any polarity in terms of intercellular communication; they have no intercalated disks and only a few scattered desmosomes.

Mitochondria are very low in numbers in P cells and are localized in a random fashion. While similar in size to those in the working myocardium, the mitochondria of P cells have less cristae and a less well organized internal structure by comparison. These cells are devoid of t‐tubules, while the sarcoplasmic reticulum (SR), the major Ca^2+^ store within CMs, is less abundant and does not show any pattern of distribution or a clear relationship with sarcomeres 58. These selected features are graded in the intermediate population, somewhat limiting their use in terms of chamber‐specific categorization.

Regarding the AVN myocytes, these are similar in structure to SAN myocytes but an even more complex classification of myocyte subtypes exist 59. The different AVN myocyte subtypes may represent transitional phenotypes between pacemaker, conduction, and working myocardium 60.

### AP Morphology

#### Resting Membrane Potential

An essential feature of nodal cells is the presence of spontaneous depolarization during phase 4 of the AP. This automaticity is absent in cells of the working myocardium and is crucial for the pacemaker properties of this region of the heart. Nodal cells do not have a resting membrane potential and their diastolic potential is better described quantitatively by its maximum diastolic value (MDP) and the rate of depolarization. In isolated human SA node cells, the MDP is approximately −60 mV, with an intrinsic beating rate of approximately 70 bpm 61. The diastolic rate of depolarization is approximately 50 mV/s 61. The major currents controlling depolarization rate are *I*_f_ and *I*_NCX_ while *I*_Kr_ may be important for the MDP. HCN1 and HCN4, the ion channels contributing to the *I*_f_ pacemaker current, are highly expressed in nodal myocytes whereas their expression is low or absent in the working myocardium 62, 63.

Exact figures for human AV node cells are not available and, given the heterogeneity and number of cell types in this region, performing a quantitative analysis is difficult. It is generally accepted that the AVN pacemaker cells are similar to P cells in function with AP morphologies in rabbit and guinea pig AV node cells similar to those reported for SAN 64

#### Depolarization Phase

Membrane depolarization is quantified as maximal upstroke velocity (*V*_max_). *V*_max_ in human SAN cells has been reported to be approximately 4.5 V/s. This is slightly higher in rabbit SAN cells (8–12 V/s) 65, 66. AP amplitude (APA) is approximately 80 mV 65. The upstroke of the AP is due to the activation of *I*_CaT_ and *I*_CaL_. *I*_CaT_ is poorly represented in working myocardium and may be a good index of nodal activity. *I*_Na_ is very small or absent and NaV1.5 is significantly reduced or absent in nodal myocytes 60. In humans, Cav1.3 and Cav3.1, two subtypes of calcium channels, are preferentially expressed in nodal cells 63.

#### Early Phase of Repolarization and Plateau (Indices of Triangulation)

Nodal cells have a relatively long AP compared with the surrounding atrial myocardium with a clear plateau, although they usually lack an early repolarization phase 67. APD50 and APD90 have been reported having values of approximately 100 and 150 ms, respectively 65. This longer plateau has been ascribed to a robust *I*_Ca_ and a lack or reduced *I*_K1_ 63, 66, which contributes to a more triangular shape of the AP in nodal myocytes.

#### Repolarization Phase

Repolarization of nodal myocytes is the consequence of the inactivation of *I*_Ca_ and activation of outward potassium currents. As mentioned above, *I*_K1_ is small and this contributes to a slow declining phase. TREK channels have been identified in human hearts but their role and chamber‐specific distribution still need to be determined 68. The two commonly used indices of AP morphology, APD50/APD90 and APD90‐APD50, are approximately 0.6 and 50 ms, respectively 65.

### Calcium Transients

Calcium regulation in nodal myocytes differs significantly from the working myocardium. Given their poor contractile function and the lack of the structural organization that regulates local calcium‐induced calcium‐release (CICR) in working myocardium, it seems that the role of calcium in nodal cells is primarily a pacemaker function (Ca^2+^ clock 69), where calcium release from the SR becomes the trigger, not the consequence, of membrane excitation. For this reason, SR calcium release precedes fast depolarization. SERCA2a and RYR2 expression is low in nodal myocytes, whereas NCX1 has an important role in sarcolemmal calcium fluxes and is robustly expressed 63, 66.

### Conduction

At the centre of the SAN and AVN, cardiac conduction is slow. This is due to the almost complete absence in the expression of the high conductance connexins Cx40 and Cx43. Most connexins expressed in nodal myocytes are Cx45, which display low conductance properties 63, 70. The expression pattern of connexins, however, is graded within the node and can vary from cell to cell.

### Molecular Markers

A number of transcription factors (TFs) are important for nodal and conduction myocyte specification in the embryo. Among these, TBX3 is an important transcriptional repressor in small rodents and fish 71, 72. While its forced expression leads to the development of nodal myocyte features 72, 73, whether its expression can be a solid marker in adult nodal myocytes is not known. SHOX2 is another transcriptor factor essential for SA node development 74 and has been used to select nodal‐like iPSC‐CMs using genetic probes 8. Again, whether SHOX2 is a robust marker of nodal myocytes in adult human hearts is unclear. Similarly, ISL‐1 has been recently reported to play an essential part in nodal and conduction system development, but its expression is strongly reduced in the adult heart 75.

## Conduction Myocytes (Purkinje Cells)

These are the myocytes found in the specialized conduction system (Purkinje fibers).

### Morphology

Human Purkinje cells are cylindrical in shape and larger than ventricular myocytes (VM). Similar to VM, they are connected end‐to‐end via well‐developed intercalated disks but they lack the organized myofibrillar and sarcomeric structure typical of the myocytes of the working myocardium 76.

### AP Morphology

Data on the AP of Purkinje cells have been acquired predominantly from animals and it has been assumed that canine Purkinje fibers are very similar to human 40. Only very recently, a study has described human undiseased Purkinje fibers showing some quantitative difference with canine cells 77.

#### Resting Membrane Potential

MDP in these cells is more negative than ventricular cells, reaching values of around −90 mV. An important feature of these cells is the spontaneous depolarization at rest despite the presence of a robust *I*_K1_ [78]. High levels of HCN channels are thought to be responsible 40, 78; an analysis in human tissue shows larger amounts of HCN1 and HCN4 expression in Purkinje cells compared with VM, but lower than atrial myocytes (AM) 53.

#### Depolarization Phase

The depolarization phase in Purkinje cells is extremely fast, with values around 400 V/s 77. This is produced by a robust *I*_Na_ but also *I*_CaL_ and *I*_CaT_ 79.

#### Early Phase of Repolarization and Plateau (Indices of Triangulation)

APs from Purkinje cells have a clear plateau that has lower values compared with ventricular cells 40, 77. This has been ascribed to a smaller amount of *I*_CaL_ in these cells 40. Recent work has shown that human Purkinje cell APs have a smaller spike and display significant similarities to ventricular APs compared with findings in canines 77. Human Purkinje cells express CaV1.2, CaV1.3, and CaV3.1. *I*_to_ in Purkinje cells is slower in recovering and induces a rate‐dependent notch which may be due to lower expression of KChip2 and Kv3.4 channel subunits 53. The expression of HERG and KvLQT1 channels is similar to VM in humans. There is currently no information available on *I*_Kr_ and while canine Purkinje cells demonstrate a robust *I*_Ks_ current, this is small or absent in rabbit cells, rendering it difficult to make any assumptions about human cells 80, 81. The AP duration is large (APD50 ∼220 ms; APD90 ∼300ms at 1 Hz) 77, possibly due to a larger persistent *I*_Na_ 40.

#### Repolarization Phase

Repolarization in Purkinje cells is slower than in ventricular cells and may be due to lower *I*_K1_ given that the expression of Kir 2.1 is comparably reduced 53, 77. APD50/APD90 and APD90‐APD50 are approximately 0.7 and 80 ms, respectively 77.

### Calcium Transients

There are no data available for calcium transients in human Purkinje cells. However, there is a low expression of all the major calcium handling proteins, including SERCA2a, NCX1, and RYR2 53. Furthermore, these cells have robust *I*_CaL_, although less than ventricular cells, and substantial *I*_CaT_ 79, 81.

### Conduction

Conduction velocity is extremely high in Purkinje fibers at 2–3 m/s 83. As expected, these cells express large amounts of Cx43 but also Cx40, differentiating Purkinje cells from neighboring ventricular cells. The expression of the low conductance Cx45 is minimal in Purkinje cells 53.

### Molecular Markers

Purkinje cells have a molecular signature more similar to atrial cells with several genes equally increased compared with ventricles (*Cav1.3, Cav3.1, Cavα2δ2, Cx40, Kv4.3, Kv1.3, Kir3.1, MiRP1, TWIK1, TASK1, HCN1, and HCN4*) and decreased compared with ventricles (*Cx43, Kir2.1, and Kir6.1*) 53. Distinct from both AM and VM, KChip2 is almost absent in Purkinje cells 53. Another noticeable difference with cells of the working myocardium is in calcium cycling genes, with *IP3R* increased and *SERCA2a, NCX1*, and *RYR2* downregulated in Purkinje cells.

## Atrial Myocytes

These are the cells of the working myocardium found in the two atria. Because the atria are readily accessible during many clinical investigations, several studies have been performed on relatively normal isolated human AM from the early 1980s 84. Human AMs are usually isolated from the atrial appendages as these are easily accessible and often resected during cardiac surgery. It is important to consider patient‐specific and anatomic regional variability 38, 85 as myocytes from different atrial regions have quantitative differences in structure and function (Fig. 1).

### Morphology

Human AM are cylindrical, striated cells with dimensions of 120 μm length and 10–15 μm diameter 86, 87. Atrial cells are often bi or multinucleated and the cytoplasm is filled with myofibrils which span from end‐to‐end. Their sarcomeres have a length of approximately 2 μm 87. MLC2A is a myofilament protein which is selectively expressed in atrial sarcomeres during development. In adult myocytes, however, *MLC2A* is expressed in both AM and VM, and it is, therefore, a poor index of atrial chamber specificity 55. Intercalated disks are well represented in AM. Mitochondria are abundant and variable in size while the SR is distributed homogeneously in the cells 87. Until recently, it was commonly accepted that atrial cells do not have t‐tubules but recent studies have shown that in large mammals, including humans, t‐tubules are present, particularly in larger cells 88. Their distribution and number is however less prominent compared with VM 88.

### AP Morphology

AP morphology in isolated human AM is very heterogeneous. Several types of atrial APs have been described (Fig. 2 38).

#### Resting Membrane Potential

A resting membrane potential of approximately −80 mV has been described in human isolated AM by several authors 84, 89, 90 although various values 91 as low as −55 mV have been reported 92. Healthy atrial cells, despite the expression of HCN channels and the presence of a small *I*_f_ 93 maintain their resting membrane potential and the rate of spontaneous activity is low. The higher stability of resting membrane potential compared with nodal cells has been ascribed to a robust *I*_K1_; however, this current is smaller compared with ventricular tissue 40, and this can explain why atrial resting membrane potential is slightly depolarized (by ∼7 mV) in atrial cells when compared with ventricular cells in the same setting 94. In canine atria, expression of Kir2.1 channels is lower and Kir2.3 is higher compared with ventricular cells but whether this differential expression is responsible for the lower *I*_K1_ in atria is unknown 40. Given the significant overlap of values reported, a less negative resting membrane potential is a weak parameter for atrial/ventricular discrimination and may even overlap with some nodal myocytes.

#### Depolarization Phase

Depolarization in AM is very fast (∼200V/s 96, 97) and is carried by *I*_Na_, with a strong expression of NaV1.5. APA is ∼80–130 mV 90, 96, 97. *I*_CaL_ and *I*_CaT_ are both present in atrial cells but *I*_CaT_ is smaller than in nodal cells, whereas it is not found in healthy ventricular cells. CaV1.3 and CaV3.1 are also expressed in the atria 40.

#### Early Phase of Repolarization and Plateau (Indices of Triangulation)

Broadly, atrial myocyte APs are described as lacking a plateau phase and being mostly triangular. However, because of the existence of multiple subtypes (Fig. 2), this is a simplistic assumption. Certain atrial cells show a clear plateau while others have a spike‐and‐dome configuration (e.g., 92, 96). In animal studies, it has been shown that there is a gradient from the SAN to the pectinate muscle and from RA to LA with a gradual shortening of APD and less negative MDP 98. The duration of the AP in atrial cells is considered to be shorter than both nodal and VM with values APD50 and 90 recorded at 1 Hz and physiological temperature of 25 ms and 200 ms, respectively 89, 91, 95. Other studies report larger values for both APD50 200 ms and APD90 400 ms 99. The reasons for this large discrepancy are unknown but it could be due to regional variations and experimental conditions. The more triangular plateau of APs seen in these cells is ascribed to differences in calcium currents and the robust presence of *I*_to_ and *I*_Kur_ 40.

#### Repolarization Phase

Repolarization in human atrial cells may help to discriminate between atrial and other chamber‐specific myocytes. Atrial cells in fact present two currents, *I*_Kur_ 92 and *I*K_Ach_ 100, which are very small or absent in nodal or ventricular cells and may contribute to faster and more triangular repolarization 40. Human atria express Kv1.5 which is absent in ventricles and is responsible for *I*_Kur_ 52. Atrial cells are sensitive to parasympathetic activation; 2 μM carbachol can significantly shorten APD in human AM through activation of *I*K_Ach_ 91. *I*_K1_ is present but less effective than in VM. *I*_to_ is robust and, in human atrial tissue, exclusively mediated by Kv4.3 [101]. APD50/APD90 and APD90‐APD50 are approximately 0.2–0.5 and 175–200 ms, respectively 95, 99.

### Calcium Transients

In AM, calcium transients are the result of a robust calcium‐induced calcium release from the SR. The process is very similar to that in their ventricular counterparts but most studies show subcellular differences that mainly originate from the ultrastructural differences between AM and VM. The less abundant and organized t‐tubular network and the smaller cytoplasmic volume may contribute to this effect 102, 103. In the absence of t‐tubules, calcium transients start at the periphery of the cells and expand longitudinally reaching the centre of the cells much later. This is ascribed to the large presence of nonjunctional RYRs in AM associated with poor calcium diffusion in a centripetal direction 102. As in ventricular cells, a positive calcium transient amplitude–frequency relationship is expected in human AM 94. At 1 Hz, atrial cells have a slightly higher diastolic [Ca^2+^] (∼200 nM) and a slightly delayed time to peak compared with a ventricular cell, but the amplitude and rate of decay are similar. SR calcium content is also similar despite a reduced SR calcium uptake and increased NCX function 94.

### Conduction

The two major connexin isoforms present in AM are Cx43 and Cx40, while Cx45 is expressed at very low level 40. Interestingly Cx40 is absent in human VM making the robust expression of a combination of Cx40 and 43 a discriminatory marker for human AM 104. However, this combination Cx40/Cx43 is also present in Purkinje fibers 104.

### Molecular Markers

Several studies have investigated transcriptional profiles in human atrial and ventricular tissue and found substantial differences in gene expression between the two chambers 105, 106. A more specific study analyzed the difference in the expression of 79 ion channel subunits in human atria, ventricles, and Purkinje fibers and determined that, compared with ventricular cells, atrial cells have a higher expression of *Cx40, Kv1.5 and Kir3.1, Cav1.3, Cav3.1, CaVα2δ2, NaVβ1, TWIK1, TASK1*, and *HCN4* 53. The atrial natriuretic factor (ANP) is a commonly used marker but its specificity for atrial tissue has been questioned 55 while several other genes are preferentially but not exclusively expressed in AM 56, 106. Sarcolipin has been proposed as a specific atrial marker in humans 107 and has been recently used to guide atrial cell selection in iPSC‐CMs 8, 9. Serpine1 and ltbp2 have been proposed as atrial specific markers in large animals 56; in human ventricles ltbp2 expression is very low 108, but whether this can allow discrimination between atrial cells and ventricular cells in humans is not known. The orphan nuclear receptor TFs COUP‐TFI and II are expressed in the human adult atria but their expression is low in the ventricles. Their expression in the human conduction system is unknown, but they have been proposed as strong atrial markers for PSC‐CMs 10.

## Ventricular Myocytes

These are the myocytes found in the two ventricles and present variability in structural and functional features between chambers, transmurally and in the apex‐base direction. The source of human ventricular tissue for isolated cell studies is usually the explanted heart during cardiac transplantation. Normal, nondiseased human VMs are very difficult to obtain as they can only be produced from large intra‐operative biopsies. Several studies have been performed on unused donor hearts 109 but whether these are truly normal has been questioned 110. More commonly, studies have been performed on “non‐failing” ventricular tissue removed during valve surgery. These samples are often portions of the heart (e.g., papillary muscles, trabeculae, etc) which may have peculiar electrophysiology and not necessarily representative of the rest of the ventricles. For this reason a solid understanding of the human normal ventricular myocyte is lacking and the details reported below are often derived from, or supported by, large animal studies.

### Morphology

VM are large rod‐shaped cells with lengths around 100–150 μm and diameter of 15–25 μm 111. They can be bifurcated and show a very organized structure with a thick, regular t‐tubular network, and aligned sarcomeres of approximately 2 μm in length 112. Ventricular cells express both MLC2A and 2V, with MLC2V specific for ventricular cells 55. Ventricular cells are often bi‐ or multinucleated and possess a large number of well spaced and homogeneous mitochondria which are distributed along the myofibrils and immediately below the cell membrane 113. Intercalated disks are extremely well developed in VM 112.

### AP Morphology

The AP of VM is characterized by very fast depolarization, a stable and long plateau and rapid depolarization. Its shape and duration, however, differs across the ventricular wall, with midmyocardium areas showing greater AP duration. Subepicardial APs have a spike‐and‐dome configuration that is absent or much less pronounced in subendocardial cells (Fig. 3 40, 114).

#### Resting Membrane Potential

A MDP of −80/–85 mV is generally described for human VM, only a few mV more negative than atrial MDP 39, 114, 115, 116, with no significant variation described in cells from different parts of the wall 115. Ventricular cells do not show spontaneous depolarization and automaticity during Phase 4 of the AP, owing to the robust presence of *I*_K1_ 40, 114. *I*_f_ is absent and HCN expression is very low in normal ventricular cells 116.

#### Depolarization Phase

Ventricular cells depolarize extremely rapidly (*V*_max_ in epi and endocardial cells of ∼200 V/s and in midmyocardium ∼300 V/s 40, 115), with an APA of ∼100 mV in all regions 114, 115. Depolarization in ventricular cells is carried out predominantly by *I*_Na_, while *I*_CaL_ is the main calcium current in these myocytes, expressing low levels of CaV1.3 and CaV3.1 [53].

#### Early Phase of Repolarization and Plateau (Indices of Triangulation)

The shape and duration of ventricular cell depolarization is strongly dependent on their location in the wall (Fig. 3 [39]). Epicardial cells show a very clear spike‐and‐dome configuration which is absent in endocardial cells 40, 115. These differences can be ascribed to different amounts of *I*_to_ which is larger in subepicardial cells 117. The presence of larger amounts of Kv1.4 distinguishes ventricles from atria 53, although is found in comparable levels in Purkinje fibers. Large *I*_Kr_ and *I*_Ks_ currents counterbalance *I*_Ca_ in ventricular cells while *I*_Kur_ is absent. Kv1.5 can be observed at the intercalated disks of ventricular cells but is absent in the longitudinal cell membrane 118. The long plateau of ventricular midmyocardial cells has been ascribed to reduced *I*_Ks_ and larger persistent *I*_Na_ 40. AP duration varies across the wall with subendocardial cells having the shortest and midmyocardial cells the longest AP duration. Values for APD50 and 90 are 200 and 270 ms in the epicardium, 300 and 400 ms in the midmyocardium, and 200 and 270 ms in the endocardium 39; however, longer APD90 values of 350 ms, 440 ms, and 330 ms for the epi‐, mid‐ and endocardium have been reported 115.

#### Repolarization Phase

The rapid repolarization of ventricular cells means that their APD90–APD50 is shorter compared with atrial cells. This is due to a robust presence of *I*_K1_ as previously mentioned, which can also be responsible for a more negative MDP 40; Kir 2.1 is strongly expressed in ventricles 53. APD50/APD90 and APD90‐APD50 are approximately 0.65–0.85 and 50–130 ms, respectively 39, 115.

### Calcium Transients

Calcium regulation in human ventricular CMs is a sophisticated function that involves the tight control of SR calcium release via the activation of voltage‐gated calcium channels in the sarcolemma. The structure of the ventricular CM t‐tubular network is crucial for the efficient coupling of excitation and calcium release. While data on calcium handling in healthy human VM are unfortunately scarce; experiments performed in nonfailing cells found a low diastolic calcium concentration (∼100–150 nM) with an average transient amplitude of ∼800 ± 200 nM 119, 120. Calcium transient decay is rapid, at a rate of ∼4.76 s^−1^, with approximately 75% of calcium removal attributed to SR uptake 120. While important calcium handling proteins such as RYR2 and phospholamban are abundantly expressed, there are gradients of other proteins across the ventricular wall, with SERCA2a more highly expressed in the epicardium 53.

### Conduction

VM do not express Cx40, and the very fast conduction in ventricles is ascribed almost exclusively to the high expression of Cx43 [104].

### Molecular Markers

Studies that looked at the transcriptome of ventricular vs atrial myocardium have been mentioned above. In terms of ion transporter‐ forming proteins, Kir2.1, RYR2, phospholamban, and Kv1.4 are highly expressed in ventricles 53. MLC2V is the most commonly used ventricle‐specific marker 122, as it is not present in the atria 123. However, its expression starts early on during development and as such its presence does not indicate that the cells have developed fully mature ventricular myocyte features. In addition, MLC2V is predominantly expressed in the right ventricle, while its expression may be low in the left ventricle and outflow tract 54. Expression of S100A1, a calcium binding protein that localizes to z‐disks and the SR, is significantly higher in ventricular cells compared with atrial cells 56; however, it is expressed in atria during development and therefore is a weak marker of chamber specificity. The homeobox protein IRX4 is a powerful marker of ventricular specificity in the murine heart 123, 124, although is not uniformly expressed throughout the chamber and is absent in the outflow tract 123. Whether this expression pattern is similar in the human heart is currently unknown.

## Quantitative Criteria to Determine Chamber‐Specificity

Table 2 contains a summary of the structural, electrophysiological, and molecular markers described above along with a color‐coded scoring system assigned to each parameter to indicate their discriminatory power. Blank cells indicate no information is currently available. Discriminatory power (1 to 3 points) was assigned based on whether the parameter was shared by three chamber‐specific myocyte types (1 point), by two myocyte types (2 points), or was unique for a particular myocyte type (3 points). Multiple values are provided for some parameters where there is disagreement in the literature.

**Table 2:** Criteria for chamber specificity of cardiac myocytes

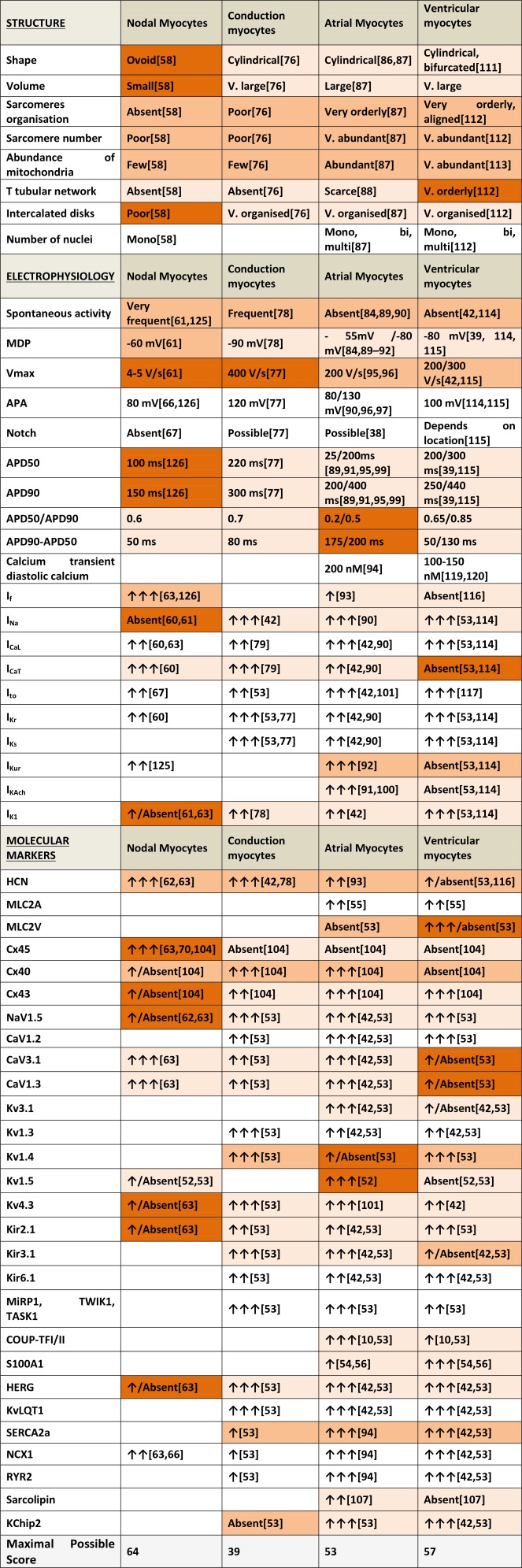

**Figure 4:**
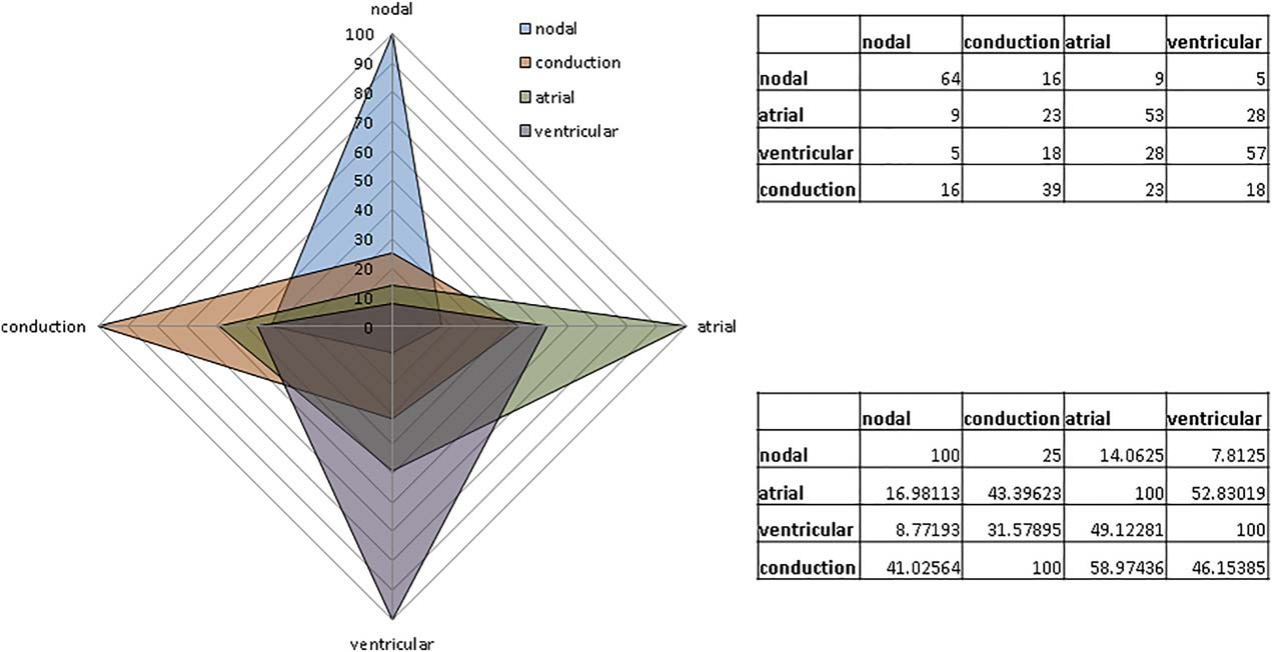
Radar chart showing the significant overlap of “specific” features when scores from the four myocyte types for each chamber‐specific template are overlaid. Nodal and ventricular myocytes (VM) present the most distinctive differences when compared, while AM and VM overlap the most. Conduction myocytes present equal overlap with the other three types. Upper table: absolute values. Lower table and chart: values are percentage of total.

With this scoring system, features from the four stereotypical myocyte types are compared using the parameters described above (Fig. 4). As one might expect, significant overlap exists as these features are rarely exclusive to a single cell type. The greatest difference is to be seen between nodal and VM where there is limited overlap not only in scoring but also physiological function. By contrast, AM and VM overlap substantially, demonstrating that determining their chamber specific phenotype requires a much more accurate and complete characterization to allow discrimination between the two cell types. Conduction myocytes appear to occupy an intermediate position, displaying substantial overlap with the other three groups.

What is clear is that there is little evidence to support the use of a single property or identifying marker to distinguish a CM as originating from one chamber or another, but rather that small differences in a wide variety of properties summate to produce these functionally distinct cells. It is on this basis that we feel that it is only correct to say that a cell of a particular chamber type is one which displays the full phenotype normally ascribed to cells of that origin. Such an approach would help support the effectiveness of PSC‐derived CMs in the applications we have discussed here, ensuring that pharmacological targets of interest are supported by a physiologically relevant context as well as carefully assessing differences in all areas between PSC‐CMs and native myocardium.

While phenotypic characterization is often qualitative in nature, we propose that the use of a scoring system such as described above offers a semiquantitative system for assessing the similarity of PSC‐CM preparations to adult CMs from different chambers of the heart. Assessed parameters in PSC‐CMs can be given a score (0–3) based on the discriminatory power for that parameter in the anatomical region they most closely match and then compared with our template for adult human CMs, allowing for a robust assessment of whatever chamber bias the cells may display. Achieving a complete parity with this adult human cardiac chamber template may not be a realistic goal or a necessary requirement for certain applications, but it does provide an evidence‐based, benchmark against which to objectively assess the fidelity of cardiac differentiation in PSC‐CMs.

By way of example of the utility of this approach, we have performed an assessment of both the commonly described features of generic iPSC‐CMs as well as several studies that reported attempts to guide iPSC‐CM differentiation toward one particular chamber phenotype. We have scored all the parameters reported in articles against the property of each chamber from Table 2. Where a parameter has not been measured, the score for that parameter is 0.

**Figure 5:**
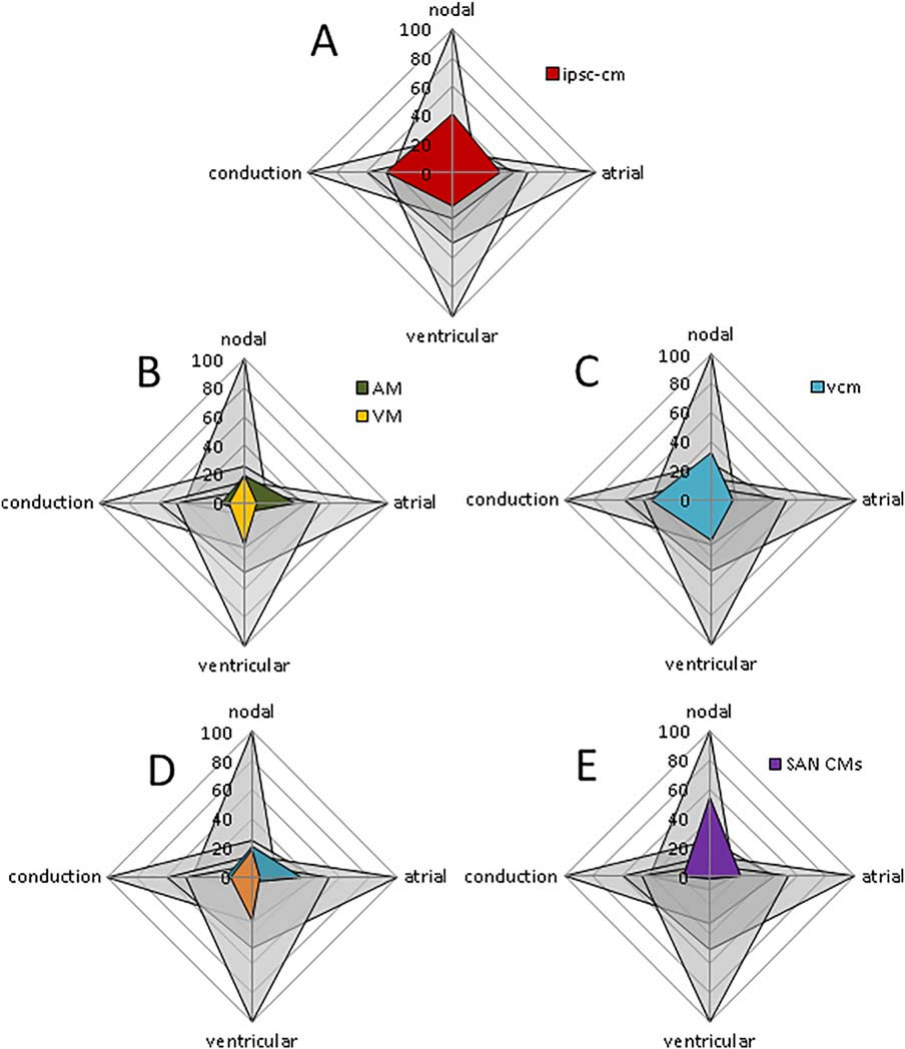
Comparison of chamber‐specific properties of various induced pluripotent stem cell (iPSC)‐cardiomyocyte (CM) lines. **(A):** Data from global analysis of recent literature 28 are used to build the profile of human iPSC‐CMs in terms of chamber specificity. **(B):** Data obtained from Devalla et al., where atrial differentiation was induced by retinoic acid and atrial myocytes (AM) were obtained 10. The radar chart shows a predominant atrial differentiation in myocytes obtained with this strategy compared with ventricular myocytes (VM); however, the demonstrated degree of similarity to adult myocytes is still low and a more detailed characterization is required. **(C):** Data obtained from Weng et al., where a method to obtain a high percentage of ventricular‐specific CMs from PSCs is reported 11. Despite a predominant ventricular over atrial axis, several features of such specific phenotype are still lacking. In addition, the cells obtained have several features of cells from the nodal/conduction system, well over the normal overlapping area. **(D):** Data obtained from Josowitz et al., where AM were sorted using red fluorescent sarcolipin (red high). The remaining, non‐fluorescent myocytes (red low) were considered VM 9. **(E):** Data from Protze et al. showing an improved method to select PSC‐CMs with features of SA node cells 36. Abbreviations: AM, atrial myocytes; CM, cardiomyocyte; iPSC, induced pluripotent stem cell; SAN, sinoatrial node; VCM, ventricular‐specific cardiomyocytes; VM, ventricular myocytes.

Figure 5A represents human iPSC‐CM properties as described in a recent, extensive review of the subject, plotted against the four adult chamber types 28. When analyzed in this way, iPSC‐CMs do not show any clear bias toward a particular chamber phenotype, mainly occupying the common ground shared by all four cell types. Of the four chambers, nodal features are the most predominant in these cells, although this is mainly due to their irregular structural morphology and high level of automaticity. These features in particular are as much representative of the relatively immature nature of iPSC‐CMs relative to the adult cells we are comparing to rather than any true dedication to a nodal phenotype, an issue we will discuss below.

A variety of strategies aimed at inducing more chamber‐specific properties in PSC‐CMs have been described in the literature. For example, Devalla et al. induced atrial‐like features in ESC‐CMs by applying retinoic acid during differentiation 10. This resulted in a number of changes driven by the COUP‐TF family, including an increase in the expression of atrial specific ion channels such as Kv1.5 and Kir3.1, greater *I*_Kur_ and *I*_KAch_ currents, as well as subsequent AP changes. Figure 5B is a representation of the phenotypic features of the “atrial” and ventricular cells presented in this report as scored by our system. Retinoic acid treatment was clearly successful in shifting the main axis of these cells toward an atrial phenotype, and the two cell groups described appear significantly distinct. The poor morphological features of these cells however and the lack of other information on other parameters mean this analysis is still incomplete and more data would be required to determine whether these cells fully possessed the features of atrial cells after retinoic acid treatment. As Devalla et al. themselves discuss, the strength of PSC‐CMs over the use of non‐recombinant cell lines overexpressing chamber‐specific targets of interest is that they provide an important context to these targets that is representative of human physiology. As such, approaches which only enhance the unique or therapeutically relevant aspects of cells from a particular chamber are still lacking, and it is important to consider the global properties of these cells when trying to recreate a truly chamber‐specific phenotype.

Another example of such chamber‐specific manipulation can be found in ref. 11 where a chemical protocol for specific ventricular specification of cardiac myocytes derived from PSC is reported. Figure 5C shows that, using the radar graph tool described above, a clear tendency toward a ventricular rather than an atrial phenotype is demonstrated. While the authors successfully demonstrated the presence of robust calcium handling that was responsive to isoproterenol as well as functional *I*_Na_, *I*_Ca_, *I*_Kr_, and *I*_KATP_ currents, the lack of organized structure, spontaneous activity, and a slow *V*_max_ suggest that these cells recapitulate many features of myocytes of the nodal/conduction groups and more work would be needed to demonstrate that these cells represent the ventricular phenotype fully.

In other studies, the idea of sorting myocytes using chamber‐specific markers has been exploited. One example is shown in Figure 5D. Josowitz et al. showed that, using red fluorescent sarcolipin, it is possible to select myocytes with more atrial‐like features 9. This can be clearly seen with the radar graph (Fig. 5D), although the overlapping of red myocytes with the atrial area is still limited for reasons similar to those indicated above. A similar approach was used by Chen et al. 8. Recently, Protze et al., have shown a robust protocol to select iPSC‐CMs with features of nodal myocytes 36. Owing to a very detailed, multifaceted characterization, the SA node specificity demonstrated in this study is convincing and can be easily visualized in Figure 5E.

While this approach gives an immediated visual comparison with the four chambers, it has some limitations: first, it assumes that studies have analyzed all the parameters described as chamber‐specific. In many studies performed so far, many of these parameters have not been measured, reducing the impact of the strategy. Second, because of lack of fundamental knowledge of what parameters are more or less important for chamber‐specificity, all parameters are weighted equally. Future experiments are required to refine the parameters and to determine a possible ranking and weight system, to be incorporated in the tool described here.

As discussed previously, PSC‐CMs are often described as having an embryonic‐fetal phenotype, however, the data used in this system is based on that known for adult CMs. As such, the maturity of PSC‐CMs will affect their scoring as much as their specification. To what degree these two aspects are interrelated is an important question, although beyond the scope of this article. It is, therefore, likely that strategies to enhance general PSC‐CM maturity, reviewed extensively here 127, will be as valuable in developing chamber‐specific CMs as those directing the development of chamber‐specific characteristics.

## Conclusion

CMs from different regions of the heart differ substantially but also overlap in many aspects, making their categorization a difficult task. The crucial effort to obtain PSC‐cardiac myocytes that reproduce specialized features of the adult human myocardium cannot ignore the issue of chamber‐specificity as the presence of only common features limited to generic cardiac contractility and electrophysiology properties are insufficient to justify the use of the cells as an alternative to existing models. The tool to assess chamber‐specific criteria provided here is a step in this direction and provides a more solid ground for further applications of PSC‐CMs in cardiovascular research.

## Author Contributions

C.K. and C.M.N.T.: ideas and concepts described, the design of the work, the analysis of the literature, the writing and the reviewing of this manuscript. C.K. and C.M.N.T. contributed equally to this article.

## Disclosure of Potential Conflicts of Interest

The authors indicate no potential conflicts of interest.
